# Larger Countermovement Increases the Jump Height of Countermovement Jump

**DOI:** 10.3390/sports6040131

**Published:** 2018-10-26

**Authors:** Alberto Sánchez-Sixto, Andrew J. Harrison, Pablo Floría

**Affiliations:** 1 Department of Sports, Centro de Estudios Universitarios Cardenal Spinola CEU, Bormujos 41930, Spain; 2 Biomechanics Research Unit, University of Limerick, Limerick V94 T9PX, Ireland; drew.harrison@ul.ie; 3 Physical Performance & Sports Research, Universidad Pablo de Olavide, Sevilla 41013, Spain; pfloriam@upo.es

**Keywords:** jumping, force plates, kinematics, kinetics, performance, basketball

## Abstract

Simulation studies show that jump performance can be improved by increasing the depth of countermovement. The purpose of this study was to determine how modifications to the depth of countermovement lead to changes in jump height and the biomechanical parameters related to center of mass displacement and force application. Twenty-nine competitive males participated in this investigation, performing nine countermovement jumps using a self-selected, a deep, and a shallow crouch position. Jump height and relative net vertical impulse were greater when using a deeper crouch position, compared to the self-selected position. Force application variables did not report differences, when the deeper countermovement was compared to the self-selected countermovement; although, the shallower countermovement showed higher values in force application parameters. The deeper countermovement jumps achieved higher velocities of the center of mass than the self-selected jumps, while shallower jumps produced lower velocities than the self-selected jumps. The results of this investigation were consistent with simulation studies, showing that deep countermovements increase net vertical impulse, leading to a higher jump height. In addition, the maximum downward velocity was higher, when the crouch position was deeper. Conversely, force-applied variables did not change when jump performance was increased.

## 1. Introduction

The vertical jump is one of the most common fundamental motor skills, and, in various sports, the capacity to jump higher than an opponent can be advantageous in competition. Thus, improving the vertical jumping ability of athletes can be an important objective for coaches, as well as strength and conditioning professionals, who often use countermovement jump height to assess athletic performance and physical conditioning. A countermovement jump is when an athlete flexes their knees and jumps as high as possible, with their arms akimbo. Many studies have examined the biomechanical parameters related to increased jump height [1,2,3,4,5,6] and the net vertical impulse, relative to body mass, has been shown to correlate with jump height [7]. Impulse refers to the amount of force applied over a period of time [8] and any change in vertical impulse is dependent on changes in either force or time. Consequently, athletes have three ways of improving jump performance: Increasing the amount of force applied, the duration of force application, or both.

In practice, the duration of force application can only be increased by increasing the center of mass displacement during the countermovement, as athletes need to apply great forces as quickly as possible, to achieve their maximum jump height [4]. Previous studies have analyzed how changes in the depth of countermovements affect vertical jump performance [6,7]. It is widely accepted that vertical jump performance is worsened, when the depth of the countermovement is restricted; however, little agreement exists on the influence on performance, when the depth of the countermovement is increased [1,2,3,5,6,7]. Previous investigations have performed simulation studies, using mathematical models of the musculoskeletal system. These investigations replicated various jumping tasks and experimental studies, where athletes performed the jumping tasks following instructions of investigators. Simulation studies have predicted an increase in vertical jump performance, with a higher squat depth [1,2]; but experimental studies, in which students and elite athletes participated, have not corroborated this finding [5,6,7]. New experimental studies could elucidate whether it is possible to instruct athletes to increase their vertical jump performance using larger countermovements than they would otherwise select by themselves, or simulation studies would have predicted [1,2]. This information could allow athletes to better leverage their capacities. Furthermore, any changes in the depth of countermovement may be accompanied by changes in other parameters, during the countermovement, such as the force applied, velocity of the center of mass, etc. These may, in turn, affect jump performance [6,7].

Increases in the magnitude of applied force should improve the jump height by increasing the net impulse. Several discrete variables, such as peak force and average force, have been used to describe the force applied during vertical jumps. The relationship between peak force and jump height has been widely researched; although results are somewhat inconclusive [4,6]. These contradictory outcomes could be explained, because previous investigations have shown that the depth of countermovement can disrupt the relationship between peak force and jump height [9,10]. Thus, the interaction between other discrete variables, related to force applied and depth of countermovement, remains unclear. Therefore, research is needed to fully understand how the changes in vertical jump technique influence force parameters.

Furthermore, greater understanding is required, regarding how biomechanical variables can be modified by varying the countermovement technique. Therefore, the purpose of this study was to determine how modifications to the depth of countermovement led to changes in jump height and the biomechanical parameters related to center of mass displacement and force application. This information could be used to determine whether increases in jump performance are due to force application or center of mass displacement variables. It was hypothesized that increase in the countermovement depth would improve jump performance, as simulation studies have shown previously [1,2].

## 2. Materials and Methods

### 2.1. Subjects

A total of 29 male basketball and soccer athletes from regional leagues participated in this investigation (age: 22.66 ± 1.37 years; height: 1.75 ± 0.05 m; and body mass: 79.79 ± 12.30 kg). No participants had experienced any musculoskeletal injury or nervous system dysfunction within 6 months before participation in this study. All participants had prior experience in jumping tasks and could perform countermovement jumps of varying depths. The study had ethical approval from the local University Research Ethics Committee and all participants provided informed consent before participation.

### 2.2. Procedures

**Figure 1:**
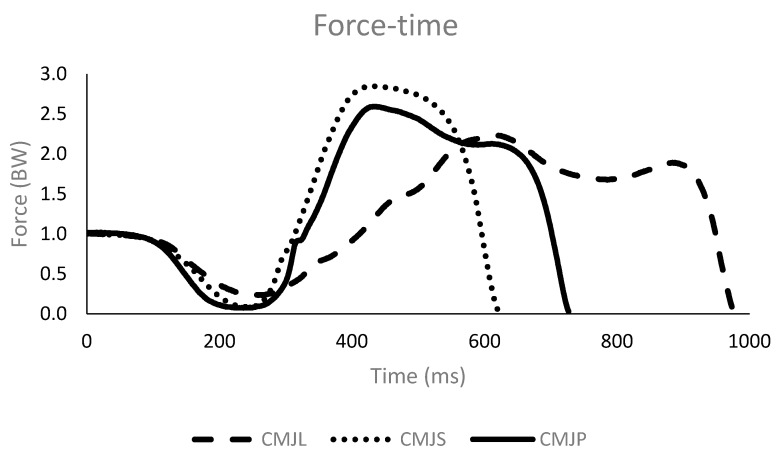
Force/time curves of the three countermovement jumps. CMJP = preferred countermovement jump, CMJS = shorter countermovement jump, CMJL = larger countermovement jump.

**Figure 2:**
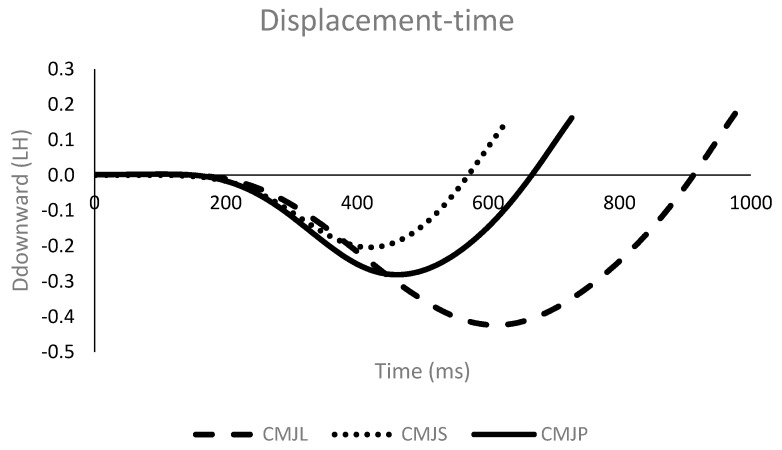
Displacement/time curves of the three countermovement jumps. CMJP = preferred countermovement jump, CMJS = shorter countermovement jump, CMJL = larger countermovement jump.

Participants were instructed to perform countermovement jumps on a force plate (Dinascan 600 M, Biomechanical Institute of Valencia, Spain), sampling at 1000 Hz. A familiarization session was completed before the jumping experiment began, during which it was verified that all participants could complete the jumping tasks at different countermovement depths and to a satisfactory level. This familiarization session determined the preferred depth of crouch for each participant (self-selected depth). Immediately before testing, all participants performed 10 min of general warm up, including 2 min of low-intensity aerobic exercise, dynamic stretching exercises, and one set of 9 sub-maximal jumps [11]. After the warm up, participants were requested to perform 3 countermovement jumps, using a self-selected crouch position, 3 countermovement jumps with a larger countermovement, and 3 countermovement jumps with a shorter countermovement, in a random order, as shown in Figure 1 and Figure 2. The standardized instructions for each participant and each jump were: “Jump as high as possible” for the self-selected countermovement jump, “Jump as high as possible with a higher countermovement depth” for the larger countermovement jump, and “Jump as high as possible with a lower countermovement depth” for the shorter countermovement jump. The following criteria were established to ensure each jump was executed successfully. A countermovement jump from a higher countermovement depth was successful, when the countermovement was at least 5 cm larger than the self-selected jump. A countermovement jump from a lower countermovement depth was successful, when the countermovement was at least 5 cm shorter than the self-selected jump. When a countermovement jump did not comply with the established criteria, participants repeated the jump. No participant had to repeat a jump more than twice. The countermovement depth was identified by displacement-time data. The displacement-time data was calculated using the impulse method [12]. Net impulse was obtained by integrating the net vertical force—with respect to time—from 2 s prior to the first movement of the participant [13], using the trapezoidal method [14]. Subsequently, the center of mass vertical velocity was calculated by dividing the net impulse by the participant’s body mass. The vertical center of mass displacement was derived by integrating the vertical center of mass velocity. To exclude the influence of weight and height on scores, all variables quantifying force were normalized to body weight (BW) and all variables quantifying displacement were normalized to leg height (i.e., standing height minus sitting height).

Participants retained the arms akimbo position from the start until the completion of the landing phase in the jumps. For every jump, each participant stood upright and stationary for at least 2 s, before initiating the jump. Three successful jumps were recorded for each jump type, with at least 2 min of rest allowed between jumps. The maximum jump height, calculated using the impulse-momentum method [14], was used to determine the best jump.

### 2.3. Analysis

The downward movement phase was defined as being from the start of movement to the instant of maximum downward displacement of the center of mass (i.e., maximum countermovement depth of the jump). The start of movement was detected by searching forward from the first intersection of vertical ground reaction force, within a predefined threshold of 1.75 times the peak residual force, during the 2-s BW averaging period. A backwards search was then performed, until ground reaction force passed through body weight [13]. The upward movement phase was defined as commencing at the moment of maximum countermovement depth of the center of mass and ending at take-off. The moment of take-off was defined as the instant in which the first intersection of vertical ground reaction force occurred, within an offset threshold. This threshold was determined by adding the average flight time (i.e., 0.4 s) and the peak residual of the offset [13]. 

Maximal height, flight height, height at take-off, and height at the beginning of the upward phase were calculated by subtracting height values from the start of the upward phase and the take-off instant. Net vertical impulse was calculated by removing the vertical impulse, exerted by gravity. The net vertical impulse was divided by the body mass to obtain the relative net vertical impulse. Minimum force was measured as the minimum value of force reached, during the downward movement phase. Force at the beginning of the upward movement phase was defined as the value of force at the instant of maximum countermovement depth. Peak force was measured as the maximum value of force reached, during the upward phase. Average force was calculated during the upward movement phase. Maximum negative velocity was the greatest downwards (i.e., negative) velocity value achieved, during the downward movement phase, and maximum velocity was the maximum velocity value reached, during the upward movement phase. Velocity of take-off was the value of velocity achieved in the take-off instant.

### 2.4. Statistical Analysis

Statistical analyses were conducted, using SPSS 18.0 software (IBM, Armonk, NY, USA). Means and standard deviations of each participant were computed for all the extracted variables (net vertical impulse, force applied, velocity, and center of mass displacement variables). Normality of the data-sets was verified, using the Shapiro-Wilk test. If the data were normally distributed, a general linear model ANOVA with a repeated measures test was used. When a significant F-value was found, post hoc pair wise comparisons of means were examined, using the least significant difference post hoc test. Significance level was set at *p* < 0.05. Since each participant performed three jumps from each countermovement depth (preferred, higher, and lower), the trial factor was included as a separate factor in the ANOVA. However, there were no interaction effects for this factor; and therefore, data are not presented in the results for the three separate trials. If the data were not normally distributed, then a Wilcoxon test was used. The magnitude of the differences between jumps was expressed as a standardized mean effect size (i.e., Cohen’s dz). The criteria to interpret the magnitude of the effect size were: trivial = 0.00–0.19, small = 0.20–0.59, moderate = 0.60–1.20, and high > 1.20 [15].

## 3. Results

### 3.1. Center of Mass Displacement Variables

The normalized mean ± SD values for the height and center of mass displacement variables are presented in Table 1, together with the statistical significances of differences between the jumps. The results show that countermovement depth had a statistically significant effect on jump performance. Both the maximum jump height and the flight height were greater, when using a higher countermovement depth (0.46 ± 0.07 m and 0.35 ± 0.05 m, respectively) compared with the self-selected position (0.44 ± 0.07 m and 0.33 ± 0.06 m, respectively; *p* ≤ 0.043; effect size ≥ 0.5). Conversely, when the countermovement depth was lower, maximum jump height and flight height (0.42 ± 0.07 m and 0.31 ± 0.05 m, respectively) were lower, in comparison to the self-selected countermovement jump (*p* ≤ 0.021; effect size ≥ 0.55). The countermovement depth was mainly responsible for differences in the center of mass displacement, during the upward phase, as no differences in the height of the center of mass at take-off were observed between the types of jump.

### 3.2. Force-Applied Variables

Results showed that the depth of countermovement also influenced the parameters which described the force applied during the jump, as shown in Table 2. The relative net vertical impulse significantly increased, with increasing depth in the countermovement. Average force values were lower in the deeper countermovement jump, when compared to the self-selected countermovement jump (*p* ≤ 0.001; effect size = 1.81). The remaining parameters related to force applied did not show differences; although, there were performance-related differences between the two types of jump (*p* ≥ 0.317; effect size ≤ 0.32). Statistically significant differences were found between the shorter countermovement and the self-selected countermovement jumps in most of parameters related to force applied. The initial force (*p* ≤ 0.046; effect size = 0.46), the maximum force (*p* ≤ 0.001; effect size = 1.54), and average force (*p* ≤ 0.001; effect size = 1.77) were higher in the shorter countermovement jumps, compared to the self-selected countermovement jump.

### 3.3. Velocity of Center of Mass Variables

Both the downward and upward velocities of the center of mass showed differences, when the self-selected countermovement jump was compared to the shorter or larger countermovement jumps, as shown in Table 3. The deeper jumps achieved higher velocities than the self-selected ones (*p* ≤ 0.004; effect size ≥ 0.67), while shorter countermovement jumps reached lower velocities than the preferred ones (*p* ≤ 0.001; effect size ≥ 0.87).

## 4. Discussion

The main finding of this investigation was that an increase in countermovement depth—at depths of at least 0.05 m—had a positive influence on jump performance. These outcomes were in accordance with previous simulation models of vertical jumps [1,2]. Other experimental studies have found that modifications to the countermovement depth lead to changes in the height jumped; although, jump performance was not higher than that achieved with a self-selected depth [3,5,7,8,16]. In this study, higher jumps were achieved, when the depth of countermovement was greater than the self-selected condition. These contrasting findings could be attributed to the instructions given to participants [3,6,7,16]. Previous studies required participants to adopt specific countermovement depths, defined by either precise knee flexion angles or exact vertical displacement of the center of mass [6,7,8]. It is likely that this compromised participants’ jump coordination sequence, by redirecting their focus on reaching the prescribed depth, rather than maximizing their effort to jump as high as possible. One other study found no differences in jump height between preferred and larger countermovements, when movement of the trunk was limited [3], suggesting that a reduction in trunk flexion limited the work and activation levels of muscles around the hip joints, during the push phase [17]. The present study used a simple instruction that slightly modified the countermovement depth, and this was enough to substantially increase the height jump. Since the measurements were randomized and taken during a single session, this investigation shows that improved execution can increase jump performance, without any improvement to physical conditioning in male, regional athletes, as simulation studies predicted [1,2].

The relationship between applied force and vertical jump performance has been widely examined in the literature but with mixed results [4,6,7,8]. The present study found higher vertical ground reaction forces without increases in jump height, when the duration of the countermovement was shorter. This suggests that force increases, in the absence of optimal range of motion, do not produce improvements to the vertical jump [5,6,7,16]. Initial maximum and average forces, during upward movement, were higher in the shorter countermovement jumps, but these resulted in lower jump heights. High levels of initial and maximum force could have been the result of the ankle and knee joints generating higher joint moments, at the beginning of the upward movement phase. A previous study found higher joint moments, when countermovement jumps were performed at 70°, compared to a 90° knee flexion angle [18]. For this reason, researchers should be careful in interpreting the forces at the beginning of the upward movement phase, because they can be highly influenced by the depth of the countermovement. Also, the individual force level of the athletes might have modified their ability to apply force during the countermovement. Future studies should evaluate whether the force level has a relationship with the countermovement depth of the jump. Finally, the average force of the upward movement phase was substantially higher at lower jumping heights. This indicates that the relationship between force and displacement variables should involve a more complex interpretation. It appears that displacement-related variables modify the force applied, but higher scores in single (i.e., discrete time point) force-related variables do not always produce increases in jump height [6,7,8]. As expected, the relative net vertical impulse was higher, when the jump height was higher, and lower, when the jump height was lower, as was shown in previous investigations [7]. These outcomes highlight that the best discrete variable with which to evaluate the difference in force application during a vertical jump—when the center of mass displacement is different—is the net vertical impulse.

This study found that a change in countermovement depth also changed the velocity of the center of mass. Higher downward and upward velocities of the center of mass, during the countermovement phase, were found when the countermovement was larger. These results were consistent with previous results that found higher jump height and joint angular velocities, when the knee flexion angle increased from 70° to 90° [18]. Moreover, peak downward velocity, during the countermovement vertical jump, has previously been related to higher performance [4]. There appears to be only one study to date which has analyzed the maximal velocity of the center of mass, during the downward movement phase, when the crouch position has been modified [16]. In this study, participants were required to perform a fast countermovement; no significant difference was found between the preferred and the larger countermovement jumps, in the peak eccentric velocity of the center of mass [16]. The instruction in the present study did not include a cue to increase the downward velocity of the center of mass; however, the results showed that this velocity was modified. It is well known that modifications to the maximum downward velocity could be used to optimize the stretch shortening cycle function [19]. Our results suggested that a combination of depth of countermovement, with a high execution velocity, could increase vertical jump performance. However, increases in the depth of countermovements, executed at low velocity, might decrease jump performance. Further studies which analyze the relationship between depth countermovement and execution velocity are needed, in order to elucidate which is the ideal combination that would increase vertical jump performance and, thereby, provide specific practical guidelines for coaches and performers.

A simple instruction which modifies the center of mass displacement could modify countermovement jump performance. Based on the results of this investigation, the instruction, “Jump as high as possible with a deeper crouch position”, maximized the jump height achieved by participants. In this sense, coaches, as well as strength and conditioning professionals, should give this instruction, to ensure that athletes perform the jump as best they can. 

## 5. Conclusions

In conclusion, the results of this investigation were consistent with simulation studies, showing that a larger countermovement induced an increase in the net vertical impulse, leading to a higher jump height. In addition, the maximum downward velocity was higher, when the countermovement depth was higher. Conversely, therefore, the center of mass displacement and velocity during the downward movement phase made a decisive impact in determining the effectiveness of countermovement jumping.

## Figures and Tables

**Table 1:** Results (mean ± SD) of height and displacement of center of mass variables.

Variables	CMJP	CMJS	CMJL	ES CMJP-CMJS	ES CMJP-CMJL
h_max_ (LH)	0.48 ± 0.08	0.45 ± 0.06 *	0.50 ± 0.08 ^#^	0.55	−0.50
h_flight_ (LH)	0.36 ± 0.07	0.33 ± 0.06 *	0.37 ± 0.06 ^#^	0.86	−0.67
h_takeoff_ (LH)	0.12 ± 0.03	0.12 ± 0.02	0.13 ± 0.02	0.03	−0.09
D_downward_ (LH)	−0.32 ± 0.06	−0.23 ± 0.05 *	−0.43 ± 0.06 ^#^	−2.02	2.47
D_upward_ (LH)	0.45 ± 0.06	0.35 ± 0.05 *	0.55 ± 0.06 ^#^	1.90	−2.16

ES = effect size, h_max_ = maximal height, h_flight_ = flight height, h_takeoff_ = height at the take-off, D_downward_ = countermovement depth, D_upward_ = vertical center of mass displacement of the upward movement phase, and LH = leg height. * denotes a significant difference between CMJP and CMJS (*p* < 0.05); ^#^ denotes a significant difference between CMJP and CMJD (*p* < 0.05).

**Table 2:** Results (mean ± SD) of force variables.

Variables	CMJP	CMJS	CMJL	ES CMJP-CMJS	ES CMJP-CMJL
Impulse_Nv_ (N/kg)	2.53 ± 0.22	2.41 ± 0.23	2.59 ± 0.20	0.51	−0.31
F_min_ (BW)	0.36 ± 0.22	0.40 ± 0.23	0.36 ± 0.18	−0.40	0.03
F_initial_ (BW)	2.35 ± 0.29	2.47 ± 0.35 *	2.36 ± 0.31	−0.46	−0.05
F_max_ (BW)	2.44 ± 0.23	2.81 ± 0.31 *	2.38 ± 0.28	−1.54	0.32
F_av_ (BW)	1.99 ± 0.16	2.23 ± 0.21 *	1.84 ± 0.14 ^#^	−1.77	1.81

Impulse_Nv_ = net vertical impulse, F_min_ = minimum force of the downward movement phase, F_initial_ = force at the beginning of the upward movement phase, F_max_ = peak force, F_av_ = average force. * denotes a significant difference between CMJP and CMJS (*p* < 0.05); ^#^ denotes a significant difference between CMJP and CMJD (*p* < 0.05).

**Table 3:** Results (mean ± SD) of velocity variables.

Variables	CMJP	CMJS	CMJL	ES CMJP-CMJS	ES CMJP-CMJL
V_min_ (m·s^−1^)	−1.15 ± 0.29	−0.95 ± 0.27 *	−1.32 ± 0.28 ^#^	−1.41	1.07
V_max_ (m·s^−1^)	2.68 ± 0.20	2.58 ± 0.18 *	2.73 ± 0.19 ^#^	0.95	−0.76
V_takeoff_ (m·s^−1^)	2.54 ± 0.22	2.44 ± 0.20 *	2.59 ± 0.21 ^#^	0.87	−0.67

V_min_ = maximum negative velocity, V_max_ = maximum positive velocity, V_takeoff_ = velocity at the take-off instant. * denotes a significant difference between CMJP and CMJS (*p* < 0.05); ^#^ denotes a significant difference between CMJP and CMJD (*p* < 0.05).
